# Coping With Human-Cat Interactions Beyond the Limits of Domesticity: Moral Pluralism in the Management of Cats and Wildlife

**DOI:** 10.3389/fvets.2021.682582

**Published:** 2021-06-11

**Authors:** Geoffrey Wandesforde-Smith, Julie K. Levy, William Lynn, Jacquie Rand, Sophie Riley, Joan E. Schaffner, Peter Joseph Wolf

**Affiliations:** ^1^Department of Political Science, University of California, Davis, Davis, CA, United States; ^2^Maddie's Shelter Medicine Program, College of Veterinary Medicine, University of Florida, Gainesville, FL, United States; ^3^George Perkins Marsh Institute, Clark University, Worcester, MA, United States; ^4^School of Veterinary Science, The University of Queensland, Gatton, QLD, Australia; ^5^Australian Pet Welfare Foundation, Kenmore, QLD, Australia; ^6^Faculty of Law, University of Technology Sydney, Ultimo, NSW, Australia; ^7^The George Washington University Law School, Washington, DC, United States; ^8^Community Programs and Services, Best Friends Animal Society, Kanab, UT, United States

**Keywords:** cats, feral cats, community cats, conservation, wildlife, trap-neuter-return

## Abstract

Although human interactions with cats are often even typically analyzed in the context of domesticity, with a focus on what sorts of interactions might make both people and cats “happy at home,” a large number of cats in the world live, for one reason or another, beyond the bounds of domesticity. Human interactions with these more or less free-living cats raise deeply controversial questions about how both the cats and the people they interact with should be sensibly managed, and about the moral imperatives that ought to guide the management of their interactions through the laws and public policies regulating both human interactions with pets and with wildlife. We review the geography of human interactions with cats living beyond the bounds of domesticity. We acknowledge the contributions made to ideas about how to manage cats by the animal protection movement. We review the tensions that have emerged over time between advocates for the eradication of free-living cats, because of the impacts they have on native wildlife species, and those who have imagined alternatives to eradication, most notably one or another variant of trap-neuter-return (TNR). The conflict over how best to deal with cats living beyond the bounds of domesticity and their wildlife impacts raises the prospect of stalemate, and we canvass and critique possibilities for moving beyond that stalemate.

## Introduction

The research literature on companion animals has a clear, understandable, and laudable focus on how and why it is that human interactions with domestic cats yield behavioral dynamics of attachment and affection and on how it might be reasonable and useful for both scientific and management purposes to measure those dynamics. A central even essential but all too often unexamined premise of this body of work is that it is both sensible and just, or if you prefer humane, for people to keep cats in their homes, more or less confined, as domestic pets (1).

Such domestication of cats, which has been under way for thousands of years (2), is deemed acceptable, even normal, because it does not completely prevent a cat from living a life of its own (3). This is in contrast to what happens, for example, when a recently captured exotic bird from Guatemala or Cameroon, say, is confined to a small cage in a domestic living room or kitchen and is abruptly and permanently cut off from the life it was living in the wild, from its own natural life (4, 5).

Indeed, the prevailing assumption in the literature about human interactions with domestic cats is that the cats can be and usually are, and certainly ought to be, content living in people's homes. While the owners for their part derive great happiness and satisfaction, even diversion and entertainment, from providing cats a place to live. There exists, moreover, a vast and profitable pet economy to ensure that when cats are kept in homes as pets they can be well-fed, given toys to play with, be cared for if they get sick or injured, and even have a decent interment after they die. One might be tempted to say that over the *longue durée* (6) human interactions with cats have made it seem not just possible but natural by now to think of cats only as domesticated pets.

What other life could cats conceivably lead except a life of contented domesticity?

## Cats and Enclosure

The truth of the matter is, however, that cats have always been and to this day remain somewhat awkward subjects for domestication. When, despite best efforts to understand what makes them content and to provide for their needs, as well as for ours in relation to them, cats stray or are forced outside the home, to live if you will beyond the bounds of domesticity, they can and in many places do survive and prosper without direct human interaction and support.

It was not until relatively recently, in fact, thanks to the widespread availability of processed cat food, absorbent cat litter, and veterinary services for spaying and neutering that completely confining cats and preventing them from spending some time outside on a daily basis became feasible. The tale has been famously told about how President Calvin Coolidge's cat had free rein to wander to and from the White House in the 1920s. In those days, Stall observed, no-one thought of confining cats indoors (7). A great many cat owners still do not impose such confinement on their pets, although they increasingly run the risk nowadays of being seen as irresponsible pet owners in need of further education (8–10).

Despite their long-standing acculturation to living with people, then, cats have retained what some would regard as an inherent biological capacity to fend for themselves. In the case of socialized pets that are allowed outdoors, the indoor/outdoor cats, this can find expression in the taking of prey even when owners keep their pets well-fed. In the case of stray but social community cats and even more so for truly feral cats receiving no human support, however, the effects of cats fending for themselves on other species can be much more controversial.

That is why it is useful in this collection of papers not to restrict our interest in human-cat interactions and the behaviors they yield to the domesticated cats eating preprepared cat food from a can or a dry food package and leaving their litter in an absorbent clay cat box. The cats that do live outside their “homes,” beyond the bounds of domesticity as we put it, and sometimes in large numbers (11, 12), raise questions about human interests in and concerns about cats that merit attention even in a set of papers primarily focused on those human-cat interactions that occur within the bounds of domesticity.

## Regulating Cats in Context

In important ways our interest in cats and their behavior, as well as our interest in whether and why and how we want to regulate that behavior, is conditioned by the context in which cats are found. Some of them lead lives that are completely wild and free ranging and some are completely tame and confined (12). Context is related to regulation. If the context is such, for example, that cats have some freedom to move around in urban and suburban places or come to inhabit open or waste spaces, so that they are living in proximity to people but are not always in their dwellings, the behavior of cats can create nuisances, threatening property and perhaps health. Complaints about these nuisances trigger a regulatory process that tries to strike a balance between the negative externalities cat behavior can cause and the positive contributions cats make to human companionship and to vermin control, notably by barn cats on farms. The norm is that the regulatory balance should be struck locally, where the costs of abating the nuisances complained about can also be considered. In addition, since the end of the nineteenth century, initially for dogs but now also for cats, local governments around the world have evolved a variety of animal control and shelter programs to implement whatever balance between the costs and benefits of regulating the negative externalities of cat behavior seems most appropriate and acceptable, given a particular local context.

Some of these local animal control programs are complex and sophisticated, and in the best of them, at the present time, the guiding moral precept, by and large, is that killing cats to bring under control the community problems cats create is both unethical and ineffective. In the USA, the National Animal Care and Control Association adopted a policy in March 2021 stating that the “indiscriminate pick up or admission of healthy free-roaming cats, regardless of temperament, for any purpose other than [trapping, neutering and returning the cats, TNR], fails to serve commonly held goals of community animal management and protection programs and, as such, is a misuse of time and public funds and should be avoided (13).”

The way this precept works in practice for the cats can vary considerably, however, from one country to another and from one locality to another, depending on how firmly the moral precept against killing impounded cats is locally held. In the aggregate and across many urban and suburban jurisdictions, it is still the case that locally managed animal control programs euthanize large numbers of cats every year, although this now occurs less frequently than used to be the case (12).

In contexts where cats are much more remote from human settlement, where cats are living in the wild and free ranging, our interest in cats and their behavior still has to reckon with the possibility of human interactions, because the food cats eat, the prey they consume, may have been accorded a protected legal status; a situation that arises most notably where cats are deemed under international, national, and state law to be an alien and invasive species and their prey are defined as native species whose continued existence can be considered essential to the survival and proper functioning of endemic ecosystems (14), although this essentialist view has given way among conservation biologists in recent years to a view of nature as inherently chaotic and disruptive.

When they live their lives in these remote contexts, particularly on offshore islands, cats arguably become part of ecological processes that can threaten the viability of other species; a view which at the extreme fuels moral panic about extinctions and tends toward biological nativism. Cats can legally be declared in some jurisdictions to be an ecologically threatening process; an environmental pest that requires abatement. Thus, the moral precept that then comes into play is typically some variant of the same one that has been applied in the past to other species defined as pests, particularly those inimical to agricultural interests, namely that pests are to be killed, possibly in large numbers and, depending on the assessed severity of the threat, even to the point of extermination (15).

The indices of programmatic success in this case ought to be measures of the extent to which threatened species then recover, an assessment that has usually been made when cat eradication programs are conducted on islands. As a matter of everyday practice in other remote contexts, however, the index of success is almost always simply the number of cats killed, even though the relationship of that number to the viability and survival prospects of threatened species is in most cases unknown and in all cases uncertain, because of multiple confounding variables that are hard to measure and interact in ways that are difficult for even the best scientists to disentangle (12, 14, 16). The status of threatened species can also be affected, for example, by the loss and degradation of habitat and by environmental change processes related to climate change. Indeed, there are important but still open questions about whether human disturbance of natural landscapes for agriculture and human settlement are more important influences on the decline in numbers of native species than the prey behavior of cats (17, 18).

## Feline Moral Pluralism

Although much has been written about the different moral precepts that might guide human-cat interactions (19), the variants theoretically on offer are not easily mapped empirically across the general population or even among cat owners. What is clear, however, is that over time, the moral imperative and obligation to treat cats humanely, regardless of the context in which they live or their ownership status, has been gaining ground.

In the case of domestic cats, it has long been the case that responsibility for the way they are treated rests primarily with documented owners, although the way this is enforced has varied across time and space. However, even semiowned and unowned cats can be very well-cared for by people who do not think of themselves as owners. Such support was provided, for example, to the free-ranging domestic cats living on public lands in the Florida Keys (20) and to cats admitted to several Trap-Neuter-Return (TNR) programs in the USA (21). In Australia, the RSPCA, reflecting the assumptions built into the Commonwealth government's Threat Abatement Plan for Predation by Feral Cats, recognizes semiowned cats as cats that are fed or are provided with other care by people who do not consider they own them. Even unowned cats of varying sociability can be indirectly dependent on humans with whom they have casual and temporary interactions (14, 22). Feral cats, at least in Australia, can thus be distinguished from domestic cats, whatever their ownership status, because feral cats are unowned, unsocialized, have no dependence on humans, survive by hunting and scavenging, and live and reproduce in the wild (23).

If the human ownership bond with cats is broken, for whatever reason, public policy generally requires that reasonable efforts should be made to renew it or to find new ownership by adoption. Cats can be cared for in shelters in the interim by attending to their health needs, for example. If the ownership bond is abused, animal cruelty legislation can sanction owners and protect the cats. The moral imperative at work for domestic cats in both these circumstances entails respect and compassion; respect for the documented ownership of cats as property and compassion for each of the individual animals treated as a pet. Essentially, the same moral imperative is at work if the concept governing human-cat interactions is one of guardianship rather than ownership, although there are legal differences between the two.

A world in which individuals own cats as their property has become, then, part of a moral universe in which the deliberate and systematic killing of large numbers of cats as a matter of public policy, whether for biodiversity conservation or any other purpose, is beyond the pale of acceptable human conduct and is widely condemned by informed public opinion, most especially but not exclusively among the documented owners of cats.

In the case, however, of cats that have no owner and are living off the land in self-sustaining populations of free-living animals, different moral considerations come into play. There is a widespread belief, particularly but not exclusively in the conservation community, for example, that there can and should be no moral bar to killing cats. This is especially so if the eradication of the cats, or their complete physical removal from the landscape short of death, if that is feasible, is premised on the moral imperative of saving other species from threats to their survival, even perhaps from extinction. The consequent willingness on the part of those who are comfortable placing a privileged value on free-living species other than cats to see large numbers of cats killed can, thus, have a major impact on the life chances of cats that live outside the home.

The resulting moral conflict over whether human interactions with cats living in remote contexts should be dominated by an urge to exterminate the cats is intense; so much so it has become an unmistakable and distinguishing characteristic of much literature that deals with human-cat interactions outside the home. In the words of McCubbin and Van Patter, “The lives and deaths of cats, big and small, are sites of contestation in the world of conservation. Beginning with small cats, the management of feral domestic felines is mired in heated conflict across the globe.” (24). Other observers have argued that “the scientific literature on this issue is mostly unbalanced in one direction or the other and the various protagonists commonly have difficulty engaging in a civil discussion of their [differences]” (12).

So, it is not unusual for some contributors caught up in this conflict to say, for example, that cats are killers, even serial killers, and that the time has come or is long past to declare all out “war” on such cats and to kill as many of them as possible, by whatever means can be shown to work, in order to end, as some would have it with an eye on recent headlines, the “pandemic” cats have inflicted on native wildlife (25). Others respond by saying that the determination to exterminate such cats stems from a “moral panic” that willfully overlooks clear evidence of alternative possibilities for treating the cats with respect and compassion and that blanket recommendations for the systematic extermination of cats are both scientifically indefensible and morally untenable (26).

The intensity of this feline moral pluralism is a relatively recent phenomenon.

## The Protective Paradigm

Until the nineteenth century, public policies for dealing with cats living beyond the bounds of domesticity rested on the assumption that such cats could and should be treated under a blunt, even primitive, and narrowly utilitarian morality. Accordingly, if they were not owned or useful, and no-one could be found to make a priority out of caring for them, cats could be quickly dispatched by any convenient means, as was true at the time for other unwanted or stray animals for which no-one had any apparent further use.

In the case of dogs, for example, Janet Davis recalls how local communities in the USA staged massive roundups in the summer, when strays were shot on sight or violently thrown into crowded wagons and later summarily dispatched at the pound (27). Other methods in use once stray pets were caught commonly included drowning, strangling, clubbing, and herding them into gas chambers, although these practices were no longer widespread by the beginning of the twentieth century. Poisoning was less common but is still in use and in some places still has its advocates for truly feral cats (28–30).

The notion that a different morality could be brought into play and that, if acted upon, would lead to different and better treatment for cats, as well as for dogs and other pets, became widespread both in Britain and the USA in the first half of the nineteenth century. It found its first major institutional expression in the USA when the New York Legislature in 1866 granted incorporation to a state animal protection society, which came to be known as the American Society for the Prevention of Cruelty to Animals (ASPCA), with police powers to prosecute abuses, and then also enacted a revised state anticruelty law. Although the initial focus was to protect from abuse horses used for haulage and transportation, it was later extended to other animals.

The account Davis provides of the subsequent evolution of the animal protection movement in the USA as a “barometer for human morality” vis-à-vis animals and as a “marker of advanced civilization” makes it clear, however, that for a long time cats were an awkward fit with the agenda for moral uplift that animal protectionists began to advance during the long nineteenth century—and for some of the reasons we have already noted. She writes, for example, that cats were conspicuously absent from the list of subjects to which early animal protectionists devoted their energies. There was certainly an interest in prosecuting individual cases of egregious cruelty to cats. Indeed, as long ago as 1641, the Massachusetts General Court had enacted a colonial statute prohibiting “any Tirranny or Crueltie toward any bruite Creature which are usuallie kept for man's use” (27).

However, when the animal protection movement came of age, most local communities did not routinely round up stray cats as they did with dogs (31). At the time, dogs were vectors for canine rabies variant, which has since been eliminated from the USA. Dogs were known killers of livestock and capable of harming people, especially children, by biting. These same considerations did not apply to cats and so cats did not become subject in the same way dogs did to local licensing, leashing, and muzzling laws. Humane groups were afraid that, if cats had to wear collars, they would strangle themselves while negotiating small and confined spaces. Muzzling and leashing requirements were thought to be impractical for “a creature that straddled the divide between wild and tame.” Although some urban residents disliked cats simply because their reproductive behaviors created local nuisances (and more cats!), the real rub for many stemmed from their belief that uncontrolled cats exerted an unacceptable impact on songbirds.

In Pasadena, California, Davis reports, hostility to outdoor cats, because they arguably were villains who took too many innocent songbirds as prey, ran so high in 1903 that the local humane society called for their extermination. “Of course, I do not mean that people should not be allowed to have cats in their own houses,” a representative of the Pasadena Humane Society argued, “but those which run wild should be put out of the way.” The moral judgment applied to cats extended further, into the realm of how relations between people should be properly ordered. The local spokesperson for the humane treatment of cats thought that her cat extermination plan would force “cat ladies” to embrace their proper place in society. “I really believe that cats can stand in the way of many marriages (for women), and I have no use for either old maids or the cats they keep” (31).

So, it took some time for domestic cats to become a major and sustained focus of the work of animal protection organizations. Davis (27) captures Katherine Grier's explanation for this:

The growth of a consumer culture of pet keeping, alongside the development of sulfonamides, parasite control, and antibiotics in the 1930s and 1940s, enabled people and their pets to live longer, healthier lives together in closer proximity. [As a consequence] attitudes toward cats, perhaps, changed the most. In the nineteenth century, some animal protectionists maligned the cat as a semiwild killer of cherished songbirds. Medical advances and new consumer products, such as cat litter in 1947, brought cats indoors. By the mid-twentieth century, dogs, cats, and sheltering dominated animal protectionism.

The social and scientific factors at work here are obviously complex (32), but the upshot of the story is clear. The moral precepts and basic operating principles according to which local communities could humanely and successfully manage cats that had strayed from home were well-established and undergoing widespread implementation by the middle of the twentieth century: respect and compassion for each animal; the impoundment of strays; sheltering and medical treatment to prevent suffering; adoption; and euthanasia as a last resort for the cats that could not be rehomed. The development and refinement of this comprehensive program is a major achievement of the animal protection movement and historians have rightly chronicled it as a story in which ideas about how people ought to treat vulnerable members of the human community have been extended to members of the animal community but without so far granting animals full moral and legal equality (31, 32).

Impressive as it was, however, this paradigm of protection still left stray cats susceptible to being killed. This was essentially because, if the cats were caught or trapped but could not be rehomed, dispatching them promptly by methods that were typically brutal was thought to be a good enough way of dealing with them, and more importantly, perhaps, because no-one had persuasively demonstrated that an alternative to killing was available.

This loophole for killing, if you will, did not sit comfortably with moral and ethical arguments that began to be made in the 1960s and 1970s that cats, as well as other animals increasingly regarded as sentient, deserved to be treated with much the same consideration as should be accorded to people (33) or that the animals had an intrinsic and individual right to be “subjects of a life” of their own (34).

Could the protectionist paradigm be extended to cats living beyond the bounds of domesticity? Could it be modified in ways that would institute programs to capture stray and unowned cats, make them healthy, remove their ability to reproduce, and then return them to their worlds to live out their own lives? Such a program would eliminate the killing loophole and reduce the need to kill stray and unowned cats to zero, or perhaps as close to zero as it is humanly possible to get.

## The Advent of TNR

These are the questions that, according to Berkeley (35), began to be asked by veterinarian Jenny Remfry and other members of the Universities Federation for Animal Welfare (UFAW) in England in the 1950s and which by the 1960s had started to receive affirmative answers, albeit based on limited local experience. Just a short time later in 1980, however, it was possible for Celia Hammond, a promoter of neutering and returning to site, to tell a national symposium organized by UFAW in London that the TNR programs Remfry had pioneered on a limited local basis could be recommended for widespread adoption.

Feral cats, Hammond maintained, could be saved from killing by making it possible for them to live in what she called “neutered colonies,” so much so that she had abandoned her earlier efforts to obviate the killing of cats by trapping, taming, and rehoming them. The TNR alternative was, she argued, “cheaper, more efficient, more humane, and - not least - more acceptable to the public.” She had observed “many hundreds of neutered colonies” with populations and social structures that had been stabilized “without any detrimental effect whatsoever.” Similar reports were made in the mid-1970s by a veterinarian in Denmark, commenting on the “reintroduction” efforts being made there by the Society for the Protection of the Cat. Thus, enthusiasm for and adoptions of TNR programs then diffused, Berkeley argues, to the USA and to many other countries (35). The earliest study of TNR done in London dates from 1978 (36).

The subsequent history of TNR is not, however, quite the unalloyed success story that Berkeley envisioned. Although a wide variety of issues surrounding the theory and practice of TNR and its impact on cat behavior has been canvassed in scholarly and professional literature (37), a literature now so large that it is difficult to track, there is no consensus over the applicability and likely success of TNR in various sorts of circumstances. This is not the place to make a comprehensive review of how divided judgments about the utility and value of TNR have evolved.

However, to make a long story short it is now reasonably clear that the success and legitimacy of TNR is not tied to its being a magic bullet that can eliminate cats living outside human sway in most contexts. Its real value lies in keeping alive cats that would otherwise be killed and in suppressing the number of outdoor cats living in and around human communities, where the vast majority of outdoor cats live. It is a way of addressing the local nuisance problems people complain about and it ameliorates some of the wildlife impacts that concern state and national policy makers. A fairly long list of preconditions has to be met to realize these benefits, and they have to be attended to with adequate resources and professionalism. However, animal protection organizations have by now distilled these requirements into manual form and have accumulated considerable experience putting them into practice (38–40). A recent analysis listed almost 40 original research papers describing and evaluating North American experience with the implementation of TNR programs (41).

Experience shows, for example, that there needs to be a welldesigned and adequately funded management program, one that is most likely to not only be implemented by a mix of voluntary individual and organizational efforts and financial contributions but can also and perhaps ideally be carried forward through at least a limited partnership with local governments and their animal control agencies. Dedicated local volunteers need to be available to trap the cats. The cats must be taken to and from a local surgery. The best TNR programs incorporate an adoption component, which has to be established and managed. Ideally, the program will monitor the status of the cats and keep good records of how the cats are faring, and this work with the cats needs to be supplemented with public education and outreach efforts, aimed primarily at helping pet owners to behave responsibly vis-à-vis their pets. TNR is best understood, then, as a methodology for managing outdoor urban and suburban cats that can and perhaps should exist side by side with other interventions more suited to remote locations.

An important key to the success TNR has been able to enjoy in the USA is that the federal government and most states classify cats as domesticated animals, which means that as a legal matter, except where federal statutes for the protection of threatened and endangered species may be implicated, the control and management of cats is primarily a local responsibility (42, 43). Given the high degree of variability in the political complexion of the several thousand general purpose governments in the USA (44), there is a strong likelihood that somewhere in the interstices of this local government system advocacy of TNR by animal protection organizations will find a foothold. If TNR is not palatable in the City of Cordova, Alabama, say, it may still find favor in the City of San Jose, California, and if not in Pecos County, Texas, then in Cook County, Illinois. Overall, a 2013 analysis showed that more than 330 local governments in the USA have acted legislatively to move forward with TNR as a preferred method for managing stray and unowned cats in their local communities (45), and a great many more jurisdictions have active TNR programs even in the absence of explicit authorizing legislation.

The structural attributes of the American federal system of government have thus combined with the vigorous exercise of animal protection advocacy to give variable political expression across the country to feline moral pluralism. In some localities, the majority of public sentiment might support a policy to kill cats that stray beyond the bounds of domesticity and cannot be accommodated by animal control and shelters. In other places, elected officials and shelter directors might respond to public opinion by avoiding killing as much as possible and may aim for a zero tally. Most localities strike a balance that is somewhere in between.

## Australia: The Effective Proscription of TNR

Federalism has yielded a very different outcome in Australia, however, where there are lots of cats, six states, two mainland territories, and some 550 local governments with various responsibilities for managing cats, as well as a good number of vigorous animal protection organizations.

Standard histories of cat management in Australia show that the impacts of cats on other species began to be observed and commented on as early as 1863 (46–48). Between that date and 1992, however, when the Commonwealth government became a signatory to the Convention on Biological Diversity and listed cats in Australia as a key threat to the conservation of native species, the realization that cats had biodiversity impacts and that they might be substantial made only halting progress. This can be traced through perhaps a dozen key publications that Denny and Dickman usefully listed (49–60). Some of this work was rudimentary, and it took some time for initial observations about the impacts of cats on birds to extend to other animals. But perhaps the most remarkable part of the story, as Denny and Dickman and others tell it (46, 47), is that even after Rolls (54) dramatically publicized the issue in 1969 (54), there was not much in the way of response. Interest in cats and their negative biodiversity impacts did not quicken noticeably in the scientific community until after 1992, eventually centering in the work of the Threatened Species Recovery Hub, a project of the National Environmental Science Program (61).

Two things are clear from this history of interest, concern and research.

The first is that, despite their apparent proclamations to the contrary, governments at all levels in Australia have never taken cat management very seriously, except to see it as an opportunity to kill pests, either by using cats as instruments for killing animals inimical to agriculture or more recently by treating cats themselves as pests and killing them in the interest of saving native species (14).

In its submission to the parliamentary cat inquiry undertaken in 2020 by the Australian House of Representatives, the Threatened Species Recovery Hub described feral cats as being “largely unmanaged,” almost 30 years after cats were formally declared to be a biodiversity threat and after plans to abate the threat they posed were supposed to be developed, funded, and at work (62). In a separate publication in the same year, the Hub scientists described domestic pet cats as “ill-governed” (28). Nothing much had apparently changed since a landmark review, published a decade earlier by some of the same principals, said that cat management in Australia, despite a long record of apparent interest and concern, was “in its infancy” (46).

On the face of it, this long-term insouciance about the environmental impacts of cats should have created a scientific and political environment in which TNR could thrive as a potentially viable alternative to killing. Animal protection organizations for their part have long taken an interest in TNR and have been anxious to learn from and apply lessons learned from TNR experiences in other countries, particularly the USA. They have been most especially interested in trying to use TNR to manage human interactions with the outdoor cats that live on the peripheries and in the interstices of the major urban and suburban population centers where the great majority of Australians, and thus the great majority of Australian pet owners, reside. The community cat program developed and advocated by the Australian Pet Welfare Foundation is a paradigmatic exemplar (40).

The second thing that is clear, however, from the history of cat management in Australia is that since a national commitment was made to implement the Biodiversity Convention in 1992, and since subordinate Commonwealth and state legislation was then enacted to give effect to that international legal commitment, TNR has for all intents and purposes been legally proscribed in Australia (14). It is an offense in at least one jurisdiction, namely Queensland, to give sustenance to animals formally declared to be a biodiversity threat. And under long-standing animal control legislation in some other Australian states, returning cats to live their lives after they have been treated through a TNR program could be prosecuted as illegal abandonment.

Animal protection organizations, most notably the Australian Pet Welfare Foundation, have nevertheless taken the risk of launching a handful of TNR programs in these legal shadowlands, while also pleading for permission to practice TNR more openly. However, they have not so far prevailed for the most part against what appear to be increasingly entrenched perceptions about cats in Australia.

The view persists in the community of conservation biologists in Australia that TNR is simply unsuited to the environmental conditions that Australians, who save for the aborigines are themselves immigrants, have created on the continent. The descendants of the cats the first settlers brought with them in 1788 are, so the argument goes, so numerous by now, so widespread across the continent (63), and so successful at reproducing and competing with native species that the prospect of releasing them after they have been captured as part of a TNR program is unconscionable. It would also cut against the grain of the perception that Australia has made solemn commitments under international law to make the conservation of its remaining native species a top policy priority at all levels of government (14).

So, it has become, in effect, an article of faith among people outside the animal protection movement in Australia that TNR is, if you will, insufficiently Australian to be good policy and practice for Australia. This is a view that the recent parliamentary inquiry on cats in Australia summarily endorses in its report (25). The report does this without any apparent regret that an opportunity to explore TNR as a viable, locally adapted alternative to a crude blanket policy of trap and kill might be missed. This conclusion is reached notwithstanding the fact that by all accounts, both those published more than a decade ago (46) and those appearing just within the last year (28), a predominant reliance on killing cats has not so far produced much in the way of positive results for Australia's native wildlife, save in the unusually controlled conditions that can be created on some islands and behind fences (12, 64). That has been the experience in New Zealand, too (65).

A breakthrough might occur with the invention and deployment of new poison delivery systems, although that appears to be at best a fraught proposition (28, 29, 66), because of public opposition and difficulties with targeting, or with an advance in genetic engineering, but that has major problems of its own (67).

## The Avoidance of Stalemate: Reimagining Resident Species?

An objective observer might be forgiven for concluding that in Australia at least the contest between advocates for and opponents of TNR has reached a stalemate.

An effort has recently been made to set out as a hypothetical exercise the terms and conditions under which questions about whether TNR might work in Australia could be resolved through cooperation and goodwill and a research program endorsed and participated in by all sides (19). However, the new parliamentary inquiry report on cats and wildlife in Australia (25) does not embrace this idea. The people associated with the Threatened Species Recovery Hub reject it, because they continue to insist that, given Australian conditions and declared policy ambitions for native species, TNR is biologically, environmentally, ethically, and economically flawed (68). Animal protectionists are unlikely to find it attractive because it gives too little credence to good research work that they have already done on TNR, and it sanctions, unnecessarily and inappropriately so in their view, too much killing of cats.

Unless, then, there is a fundamental shift in the grounds on which both scientific and political disagreements about how to manage human interactions with cats living outside the home in Australia might be mitigated, it is hard to see how parties contending over the practice and promise of TNR can avoid a future in which they continue to throw occasional grenades at each other in the pages of academic journals and in legislative lobbies, and the winner will turn out to be whichever side can best withstand and afford the resulting political attrition.

One alternative way forward was sketched by environmental scientists in Australia and New Zealand about 50 years ago. In 1973, two ecologists studying the Maori rat (kiore), which was brought to New Zealand in the canoes of Polynesian immigrants, long before the advent of European settlers, observed that the animal was being referred to as native, even though it was introduced. It had, they wrote, “even crept into the ranks of desirable native wildlife, vying with such elite as the tuatara and saddleback for protection on select island refuges.” Could an introduced rat “with but squatter's rights aspire [to native status], and how much longer must later introductions await similar recognition?” This country, to paraphrase what they wrote, “will [not] come of age ecologically [until] Western man and his animal introductions are regarded as part of the natural environment” (69).

This prompted Carolyn King, a world-renowned student of the ecology of pest management, to say in 1990 that “It is time that the native and introduced mammals [in New Zealand] were treated in practice as *resident species* of equal status in the scientific sense (emphasis added).” This would recognize, she argued, that Europeans now live in the country but that they have become, along with the animals they brought with them, including the cat, part of “a working, evolving community….[that] will continue to evolve according to natural processes largely beyond our control” (70).

Essentially, the same point was made by Arthur Bentley who in 1978 published an analysis of the consequences flowing from the introduction of deer into south-east Australia. Being un-Australian in origin, he wrote, the deer “are considered not quite right for the country.” On the other hand, the deer living in the south-east Australian bush represented, in the late 1970s, “a valuable and irreplaceable asset,” and treating them as exotics that should be eliminated was “sheer humbug.” How could a “white exotic human,” conjuring up a “Dreamtime environment” that needed to be preserved, condemn to elimination a species he or she was never likely to see (71).

There is, in other words, some malleability to the notion of what constitutes a native species and a good deal of leverage to be had from reimagining both native and introduced animals simply as currently resident species, living alongside the immigrant settlers whose descendants are now also residents of Australia. When Carolyn King went to New Zealand from Oxford in the early 1980s to study the impacts of introduced stoats on native wildlife, she did not, however, find much interest in seeing stoats as residents, which prompted her to observe that the desire to protect the animals the stoats were eating did not adequately take account of the fact that but for human bungling and mismanagement the stoats would not be a problem in New Zealand at all (72).

## The Avoidance of Stalemate: Turning to Enclosure?

Given the zealousness with which conservation biologists strive to protect what they choose to regard as native species, and given that they have vigorously advanced a political agenda to recreate a biodiversity ideal their ancestors in the antipodes long ago abandoned, for what they thought at the time were good and sufficient reasons, the idea of now reimagining cats as residents, equal in status for scientific and policy purposes to the animals that were living in Australia in 1788, when the first white settlers arrived and brought various animals with them, may not prove attractive to conservation biologists any time soon (73).

Meanwhile, the native species they care most about remain under threat, and three decades after it began in earnest the effort to manage the contribution outdoor cats make to that threat continues, by their own admission, to languish, with bright spots only here and there. The analysis and recommendations offered up by the recent parliamentary inquiry to correct this situation have already been declared to be interesting and perhaps even in some ways promising, but in a fundamental sense inadequate (74).

The principal spur to a more determined and sustained campaign against cats was supposed to be an unimpeachable calculation, or ultra-sophisticated statistical estimation at least, of exactly how many cats exist beyond the bounds of domesticity in Australia, where they live, what they eat, and what proportion of their diet consists of native species (28). The strategic gamble on the part of researchers chasing these numbers was that the impacts of cats on native species, once they were properly quantified, would prove to be so large and so pervasive across the country that the wisdom of killing cats in large numbers—perhaps as many as two million dead cats from a vigorously prosecuted eradication conducted in accordance with the official Threatened Species Strategy (14)—would be self-evident. There is not much doubt that Australia now has better numbers about the cats who live there in various contexts, and about what the outdoor cats eat, than any other country in the world. However, as the report of the parliamentary cat inquiry reveals, there is a lot more to making socially licensed policies for cat management than the imaginative generation of good numbers about cats and their impacts. In this context, there is a quickening interest in enclosure.

There are at present, for example, only a limited number of reserves on the continental mainland of Australia in which native species are favored and from which cats are excluded, whether by fencing or some other means (64). The report of the parliamentary cat inquiry (25) endorses a Project Noah to create more such reserves, although the details of how and where that policy would be carried out, particularly in mainland Australia, what it would cost, and what relevance it might have for urban and suburban rather than bush and outback landscapes on the continent remain to be determined.

Whatever the details turn out to be, this strategic turn to make the tighter enclosure of threatened native species a featured addition to the toolbox used to manage the impacts cats have on those species is an important acknowledgment that killing cats cannot be relied upon to get the job done. The argument a decade ago was that “in the absence of any other long-term eradication programs for cats on the mainland, exclusion fencing has proved to be effective for the protection of many vulnerable and endangered species” (46). However, the evidence adduced for this at the time was anecdotal, the methodologies for effectively excluding cats and other predators, such as foxes, from fenced enclosures were unsettled, and the preference for eradication as a first resort was undiminished (46).

A decade ago, in other words, reserves where threatened species would be protected behind fences were an exceptional remedy for the cat problem and no more than a fallback from killing cats. It is hard to see the turn to featuring protected reserves as a mainstream public policy for managing human-cat interactions in Australia as anything other than a strategic retreat from cat eradication, and at the very least, it signals a growing awareness that the dividends from paying closer management attention to species at risk are probably greater than a single-minded focus on killing cats. The best numbers show that “over 300,000 feral cats are killed (in Australia) annually, with much of that effort happening outside the traditional conservation sector” (75). That is not a rate of kill sufficient to control, let alone eradicate, the “1.4 to 5.6 million feral cats in the Australian bush (depending on recent rainfall patterns through the arid zone),” to which must be added the 0.7 million living in towns and cities (28).

A second and perhaps even more radical exploration of enclosure as a way out of the stalemate that now seems to mark cat management appears in work on the management of Australian pet cats that was published just as the recent parliamentary cat inquiry was getting under way (28):

For pet cats, given enough political and public support, the available technical solutions for reducing impacts are simple; responsible cat ownership includes actions such as early age desexing, keeping pets indoors or in a securely contained outdoor area, and designating suburbs adjacent to high conservation-value areas as cat-free. Reducing the numbers of feral cats living in towns and cities is more challenging, but tighter management of refuse and sites of high food subsidy should reduce cat numbers substantially. As well as reducing cat impacts on ‘urban’ wildlife, reducing the numbers of pet and feral cats wandering at large will also reduce transmission rates of cat-dependent pathogens.

Although there is much to be said for responsible cat ownership, which has been central to the agenda of animal protection organizations for decades, it is not by any stretch of the imagination a straightforward “technical solution” to the problem of managing pet cats. Nor is it “simple.” The veterinary costs alone of fully implementing responsible cat ownership can be substantial and the ability to pay them is unevenly distributed amongst documented cat owners, which is why many of them do not incur such costs, even if they can be persuaded that it is the right thing for cat owners to do, and even if local legislation requires it.

Moreover, in Australia, as elsewhere, only a limited number of people who are the documented owners of cats live in homes capable of providing “securely contained outdoor areas” for the enclosure of their pets. One might even say that for people who live in apartments and other multifamily dwellings that is an insensitive recommendation. Tightening up the rules of enclosure for cats living “at home,” and dealing more aggressively with outdoor cats living near towns and cities, or adjacent to high value conservation areas and refuse sites, will also have substantial enforcement costs. The exact magnitude of these remains to be estimated but whatever they are they will cut against the likelihood that managing cats through more rigorous enclosure will find “enough political and public support” to achieve social license.

A decade ago, the prospect of tighter rules of enclosure for cats kept as domestic pets was barely a blip on the radar screen of conservation biologists looking for ways to protect native species. Denny and Dickman, for example, briefly observed that “the control of owned, domestic cats is an important aspect for the control of all cats on the Australian mainland,” (46) and literally left it at that.

The very much more pointed recommendation now from the principals associated with the Threatened Species Recovery Hub is that for all intents and purposes long-standing and socially accepted understandings of what it means to own a cat as a domestic pet, both for the owner and for the pet, need to be redefined or renegotiated so that all documented owners are required to sign up for, finance, and in the first instance enforce a full array of regulatory measures, the sum and substance of which is that no matter the circumstances in which they live their lives pet cats will no longer be able to roam.

The likelihood is, however, that this will be a step too far for most of the people who are the documented owners of the 3.8 million cats now estimated to live in Australian homes (28), including the farmers who still rely on them for pest control. It is not, on its face, a policy that is consistent with the welfare of the animals to be enclosed (4, 76). Also, it is an imposition on pet cats and their owners that could almost certainly be avoided if proper steps were taken, with the help of TNR, to reduce the number of cats who pose a threat to biodiversity because they do not live at home.

Between the total incarceration and the total non-confinement of cats, one imagines that there is a middle ground, so far largely unexplored in any systematic way, in which it becomes clear, much clearer than it is now, what Australian and American landscapes, and other landscapes too, would look like if the presumption that cats only belong indoors under strict human ownership and control was abandoned.

## Conclusion

We have made great strides, particularly since the 1950s, in examining carefully and coping more effectively with those human-cat interactions that occur beyond the bounds of domesticity. We know more than ever about the dynamics that shape such interactions and what their wider impacts are, especially on other species. However, this greater knowledge has not yet yielded any settled reconciliation of the different moral imperatives people think should govern our relationships with the cats that do not live at home.

Clearly, the ones that do get away, for whatever reason, and then live off the land, as they can do, exact a toll on other species, and that may cascade into ecosystem effects. What moral judgment should we make about that price, which would not have been exacted at all if settler societies had not introduced cats to new worlds, in an effort let it be said to make the settlers feel comfortable in worlds to which they were also new? Is it a price worth paying if both some of the cats and some of their prey remain alive, and continue to coevolve? Or are we morally obliged to restore the *status quo ante*: to worship, as David Lowenthal has it, at the altar of a biological purity which is to be saved at almost any cost from contamination by introduced aliens. We are still struggling to find the answers to these questions, although Lowenthal himself was quite clear that indigenous purity is neither possible nor desirable (77):

Nature and culture alike generally benefit from creative intermingling. Ex-colonial Jamaica, for example, readily domesticates what is alien. Since the seventeenth century, trees, grasses, crops and flowers brought in from the East Indies, Africa, North America and Europe have spread throughout the island. Do Jamaicans resent this riotous medley for displacing native flora? Quite the contrary; they rejoice in it as intrinsically Jamaican. They celebrate the commingled fragments of manifold ecologies enhanced by exotica from every land.

It is reasonable to infer that Lowenthal would have wished Australians could feel about their cats the same way that Jamaicans feel about their plants: that the cats have become intrinsically Australian. Years ago, he retold a story about a playwright who in the 1930s had converted a scruffy patch of New England land into a fine country estate. The playwright was visited by a preacher who congratulated his host on the beautiful place he had built, him and God together. “Yes,” the host replied, “and you should have seen it when God had it all to Himself” (77). We cannot go back, either in Australia or in any other country, to days when God had it all to himself. If we want to find places where all cats can live lives of their own in the different worlds people have made for them in different places around the globe, it will do no good to pretend that by completely enclosing cats, whether in homes or in fenced enclosures, we will have found ways to solve the cat problem that are acceptable, enduring and consistent with the nature of cats as animals.

## Author Contributions

GW-S drafted the article. JL, WL, JR, SR, JS, and PW listed have made a substantial, direct and intellectual contribution to reviewing and revising the work, and have approved it for publication.

## Conflict of Interest

PW is employed by Best Friends Animal Society, advocating for the protection of domestic cats via public policy initiatives. The remaining authors declare that the research was conducted in the absence of any commercial or financial relationships that could be construed as a potential conflict of interest.
